# Supplementary figures and images for: Dual STDP processes at Purkinje cells contribute to distinct improvements in accuracy and speed of saccadic eye movements
Source: PLoS Comput Biol. 2022 Oct 4;18(10):e1010564. doi: 10.1371/journal.pcbi.1010564 (PMC9565489; doi:10.1371/journal.pcbi.1010564)

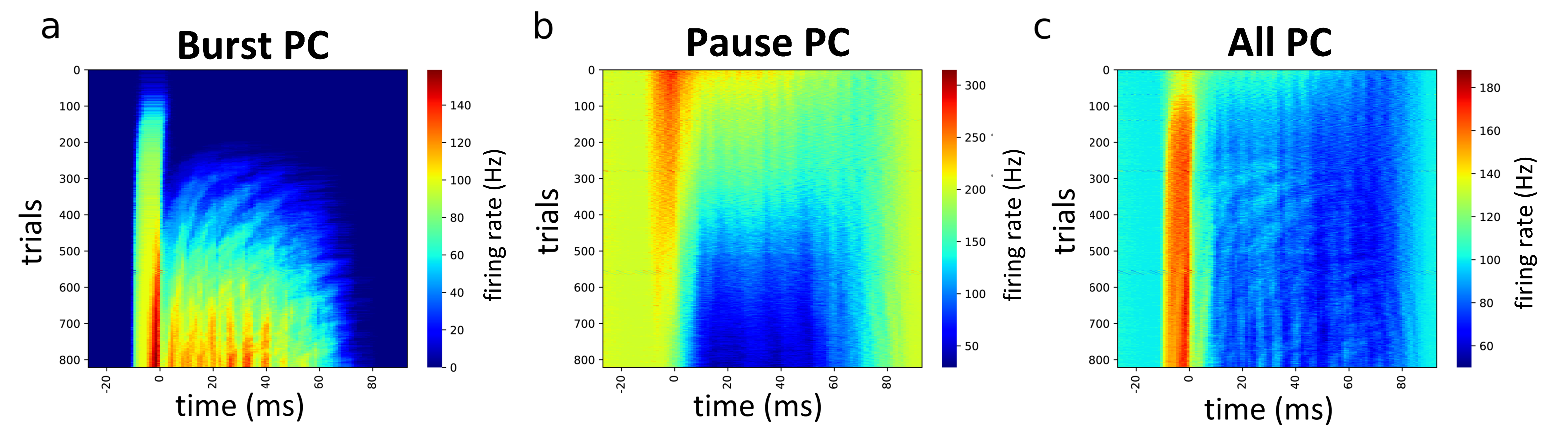

Supplement: S1 Fig — The heatmap corresponds to the mean firing rate of PC subpopulations: a) burst PCs, b) pause PCs, c) all PCs. (TIF) [file pcbi.1010564.s001.tif]

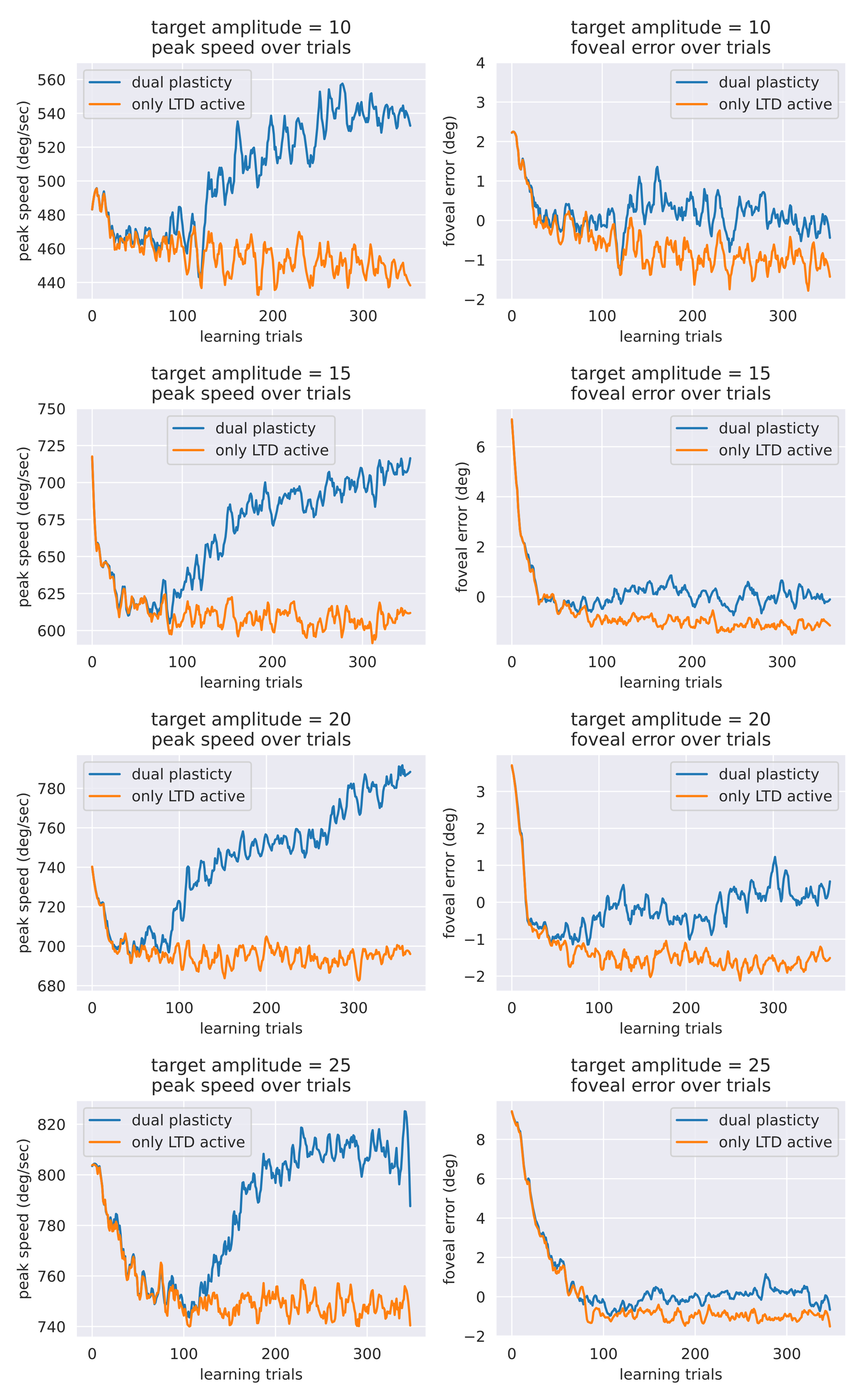

Supplement: S2 Fig — (TIF) [file pcbi.1010564.s002.tif]
