# Supplementary material for: Dual STDP processes at Purkinje cells contribute to distinct improvements in accuracy and speed of saccadic eye movements
Source: PLoS Comput Biol. 2022 Oct 4;18(10):e1010564. doi: 10.1371/journal.pcbi.1010564 (PMC9565489; doi:10.1371/journal.pcbi.1010564)
Supplement: Attachment — Submitted filename: reviewer_response.pdf [file pcbi.1010564.s004.pdf]

Reviewer #1:

The study "Dual STDP processes at Purkinje cells …" [PCOMPBIOL-D-22_00441] describes a computational model of the cerebellum that accounts for how dual plasticity processes contribute to saccade endpoint control and kinematics. They posit 2 learning rules – LTD which is error-driven, and LTP which is error-independent – as well as upward and downward modulated Purkinje cells. This cerebellar module receives target related information and sends output, via the DCN to the saccade burst generator networks in the brainstem, to influence saccadic eye movements. The upshot of their findings is that this arrangement facilitates movement optimization, enhancing endpoint control and speeds up the movements. Exploration of the substrates of this improved motor behavior showed that LTP and LTD mediate different aspects of motor enhancement in their model and they require integration between upward and downward-going Purkinje cells. I thoroughly enjoyed this manuscript, found the results intriguing, and think the study will be of interest in the field of cerebellar learning and control systems theory. Below I outline a few suggestions that may improve the manuscript.

We thank the reviewer for appreciating our work and for the precise comments, which have been very useful to improve the manuscript.

R1 - Q1. Line 301-307. The authors describe the effects on Purkinje cells of turning on and off LTP and LTD. It is not clear why turning off LTP makes PC pausers increase firing, though later the opposite is not seen when LTD is turned off. It would be helpful to provide an intuition for the reader here.

Thank you for noticing. We actually reported an erroneous description of Figure 4f . In contrast to what we wrote: *"…while the pause PC subpopulation firing rate remained constant across the training period (Fig 4f)"*, one can see a change in the pause of PC activity when LTD is switched off (difference between blue and orange traces). The change is small because the LTP parameter affects the pause of PC activity on a slow timescale. When we run the training for longer trials, the change is larger than that visible in Fig. 4f. We have corrected this mistake in the updated manuscript, Lines 403-410 in the tracked version:

"Switching off LTD caused opposite effects on the activity of burst and pause PC subpopulations, compared to those observed when LTP was switched-off. The activity of burst PCs increased above the baseline (Fig 5e) (p <0.001 with t-test, considering the max activity in 10 trials each before and after training; normality was verified for both groups of the data using kstest). On the other hand, the activity of pause PCs decreased towards the baseline (Fig 5f) (p <0.001 with t-test, considering the minimum activity in 10 trials each before and after training; normality was verified for both groups of the data using kstest)."

R1 - Q2. A little more information about the learning rules should be presented in the main text.

At the beginning of the result section (Lines 181-204 in the updated manuscript), we have added the following paragraph to explain the learning rules before the simulation results are presented. Then, a detailed mathematical formulation, and parameter tuning is presented in the Methods (Plasticity rule section).

"In this work, a given saccade is considered to be accurate if it incurs a low error, defined as the difference between the target angular displacement and the end movement displacement. We used errors expressed in degrees of rotation, but the same model can work with retinotopic or pixel difference between target and the end foveation locations (35). When the end movement displacement is higher than the target displacement i.e., overshooting movement, it incurs a positive error, while an undershooting movement incurs a negative error.

There are two main ways by which the given saccade controller can modulate the saccades. First, STDP mechanisms influence the strength of PF-PC synapses such that the PC population activity is modified. In this case the total input MF drive to the cerebellum from the superior colliculus can remain unchanged across trials. Second, the modulation of neural activity occurs upstream of the cerebellum (e.g., in the superior colliculus), and influences the cerebellum to produce a different PC output. Here, we consider the former process focusing on the effect of STDP on end errors and peak speed.

There are multiple mechanisms of plasticity at the PF-PC synapses (46). We explored the hypothesis that a dual STDP process in PCs, comprising error-dependent LTD (Equation 5.3-

5.7) and error-independent LTP (Equation 5.2-5.3), is involved in decreasing the end foveal error and increasing the peak speed of eye movements. Briefly, LTP potentiates the PF-PC synapses, and therefore it increases PC burst activity above the baseline firing rate, while decreasing PC pause. Conversely, LTD happens in response to the presence of the error signal, conveyed by the climbing fibers. Thus, it decreases the PC burst proportional to the end foveal error, while it increases the drop of PC pause below baseline. In combination, the LTP and LTD can set appropriate firing rates in both burst and pause PC subpopulations, to control the overall motor drive output from the cerebellum (sent as a speed command to the brainstem burst neurons).”

R1 - Q3. The model’s DCN output, though essential, is weakly described. I did not follow what the tuning of its output(s) are, how they influence the burst generator, nor their integration rules of Purkinje cells. I did not follow how much mixing of PC types there was in each DCN neuron. I did not follow how the speed and endpoint control are mediated by the same DCN neurons (or if so). If the PCs exert vectorial learning, do the DCN neurons exert vectorial control? If so, what are the assumptions about their directional tuning? Can any predictions be made from this model on how DCN neurons fire to control movement? what their tuning might reflect? Etc? Finally, does model include the DCN output neurons inhibitory onto the IO, as are known to exist? If not, it would be worth discussing the limitations of the model without this feedback circuit element.

We agree with the reviewer about the insufficient description of the output. Each DCN in the current model receives an equal number of burst and pause PCs. (64 number of PC per DCN divided into two equal halves of burst and pause PC subpopulations, and a total of 6 DCNs all of them causing motor output for the horizontal movements). Importantly, there are no separate DCNs to control velocity and endpoint error independently. The DCN output influences both velocity and endpoint error, by producing a cumulative motor command that persists throughout the movement. Automatically, without any hardcoding, the early component controls the acceleration (hence determining the peak speed) and the late component controls the deceleration (controlling the overall accumulated error). We updated Figure 1 to indicate the number of cells included in each of the cerebellar layer.

Our work focuses on how the PC population activity controls fast and accurate movements, without adding further complexity in the DCN circuit or nucleo-olivary loop. In the model, DCNs simply relay PC information to the brain stem burst generators, When the model is trained, the burst PC subpopulations produce an early increased velocity command, while the pause PC subpopulations produce a late decrease in the velocity command (as shown in Figure 2c). A combination of burst and pause populations (Figure 2c) can produce motor command during both the acceleration period (that determines the peak speed) and deceleration period of the movement (that determines the endpoint displacement). Hence, in the model, a phasic DCN activity reciprocal to the PC population activity during the acceleration period and deceleration period can control both peak speed and endpoint displacement. If we were to simulate the same model for vectorial control, then the DCNs would inherit the properties of the projecting PCs i.e., to have velocity encoding multiplied by the cosine of the angle between current movement direction and preferred error-tuning direction (termed as gain-field encoding in Herzfeld 2015) (although in opposite polarity due to the inhibitory nature of PC-DCN connections). However, the biological DCNs have additional connections from MFs and the nucleo-olivary loop that are not included here, potentially limiting the predictive capabilities of our model. The DCN-IO circuit is important to generate internal spatio-temporal dynamics coordinating large sets of PCs (D'Angelo 2011) and to regulate plasticity in the circuit by adding slower time constants to the learning process (Monaco et al. 2014, Monaco et al., 2018, D'Angelo et al., 2016, Smith et al., 2006). These aspects should become critical in complex multifactorial behaviors evolving over multiple spatial scales but, since this was not the case here, the absence of a more precise representation of the IO-DCN loop was unlikely to cause relevant drawbacks. We included this discussion in the main text (Lines 600-627) to clarify the model to the reader and highlight its current limitations.

"The limitation of the current model is the simplicity of different brain regions, in spite of a detailed biologically inspired construction of the cerebellar network. The model focused on how the PC population activity can be adaptively modified to influence peak speed, and end visual error while the target-related input drive to the cerebellum and the rest of the controller remains unaltered across trials. But the saccade control is composed of different regions, and especially Superior colliculus (SC) activity is implicated in determining a motor plan, that the downstream

cerebellum and brainstem burst neurons can implement (73,74). In principle, the SC activity can be altered to change the input drive (MF activity) to the cerebellum and the brain stem burst neurons, which can subsequently lead to change in peak eye speed. Notably, our modeling results do not negate the possibility that the SC influences peak eye speed by modulating its input MF drive to the cerebellum. Indeed, in Fig 4, we show that modulating the input MF drive to the cerebellum results in different peak eye speeds. However, the set of presented results are better viewed as evidence that the cerebellar STDP mechanisms can exert peak speed modulation, even if the SC/target-related input drive remains constant (see (27)). Furthermore, our future work will focus on implementing a more realistic DCN circuit. Each DCN is a hub of diverse incoming projections from MFs, PCs, and also a recurrent nucleo-olivary inhibition loop (75). Biological DCNs have additional connections from MFs and the nucleo-olivary loop that are not included here, potentially limiting the predictive capabilities of our model. Functionally, the DCNs can display unique spiking characteristics such as rebound spikes during saccades (22), that can help them sustain motor output while the inputs are extinguished. The DCN-IO circuit is important to generate internal spatio-temporal dynamics coordinating large sets of PCs (46) and to regulate plasticity in the circuit by adding slower time constants to the learning process (40,53,76). These aspects should become critical in complex multifactorial behaviors evolving over multiple spatial scales but, since this was not the case here, the absence of a more precise representation of the IO-DCN loop was unlikely to cause relevant drawbacks. Overall, it remains to be seen how the cerebellum model presented in this work shares computations with other regions, by integrating realistic circuit models of SC, DCN, and brainstem circuits.”

R1 - Q4. In the section 253-268 (approximately), on predictive control, it was hard to appreciate why reducing MF activity was used to test this. Better exposition setting up this experimental design is warranted.

The main rationale behind manipulating MF activity instead of PC activity directly is because the former is biologically plausible. In the biological circuit, PC activity is dependent on what kind of task-relevant information is conveyed by the MFs, and on how this information is transformed in the granule layer and subsequent parallel fiber, molecular layer interneurons before projecting onto PCs. Hence, we modulated the intensity of MF activity to understand if it was reliably represented at the level of PC modulation, and how it subsequently affected movement

kinematics. Our simulation reproduced the influence of PC population activity on the peak speed observed biologically (Herzfeld 2015), even though we used MF modulation to indirectly affect the PC activity. Movement kinematics would be similarly affected by directly manipulating the current-to-spike properties of the PCs and hence PC activity.

We have added this rationale in the results (Lines 322-326 in the updated manuscript)

"Note that here we modulated the MF activity to produce variation in the PC output, instead of directly modulating the PC activity by adjusting the voltage dynamics of the PCs. This protocol allows us to understand if the PC output and the eye speed can be controlled by task relevant inputs through MF afferents (projecting from regions upstream of the cerebellum) driving the cerebellar activity."

Reviewer #2:

This study investigates the neural mechanism underlying the control of saccade vigor and accuracy. They have developed a novel model of cerebellar control employing spike-timing dependent plasticity rules and shown via simulations that a control policy using a dual plasticity mechanism predicts changes in saccade accuracy and vigor. Specifically, they show that, with training, the model reduces movement error to a 10-degree target, while also increasing saccade peak velocity. They then show that these effects rely differentially on LTD (for accuracy) and LTP (velocity increase).

Generally, it is interesting to see that the simulation predicts these independent effects of LTD and LTP on accuracy and peak velocity, respectively. However, my main concern is that I am not convinced that the behavior they see in the simulation has been observed experimentally. In their model, initial saccades to a 10-degree target are slow and exhibit overshoot. With training, over many trials, overshoot errors are reduced and saccades increase in peak velocity. While there are many experimental demonstrations of error reduction with training, is there evidence of a concomitant increase in peak velocity? The paper does not clarify whether or not this is the case or any reason why we should expect to see an increase in peak velocity with lower amplitude movements. Additionally, there is no

clear rationale presented for why the cerebellum is involved in the setting and control of saccade velocity. In the points below, I explain the many reasons I am confused by their claims and ultimately left unconvinced by the validity of the model.

We thank the reviewer for the critical feedback, that helped improve the rationale and clarity of our manuscript. Particularly, we added additional results plotting the speed-amplitude and duration-amplitude relationships emergent from the simulations for validation.

R2 - Q1. The model predicts a reduction in overshoot and an increase in peak velocity with training. There is no rationale presented for why we should expect the latter.

(a) What is the rationale for why peak velocity increases with training? Importantly, the amplitude is also reduced with training. Peak velocity increases despite reductions in saccade amplitude.

(b) Again, the rationale here is not clear to me. Are the authors suggesting that peak velocity is modulated independently of the main sequence, and will increase despite reductions in amplitude?

We thank the reviewer for pointing out these important questions. Although we show that the amplitude decreases, and peak speed increases in Figure 2 the speed does not exceed the maximum values known from previous monkey experiments (Robinson et al., 1993, Fuchs 1967). Our aim was instead to show that by means of heterogeneous plasticity rules, the cerebellum can control speed to achieve/maintain main-sequence level movements even if the input drive from SC/MF remains constant. Conversely, the absence of the cerebellum causes movements that are relatively slower, with longer duration.

There is experimental evidence that the cerebellum influences/restores the speed-amplitude relationship (Robinson 1993, and Robinson 1980). In Robinson 1993, the authors inactivated the cerebellar output by blocking the fastigial nuclei using muscimol injections (see Robinson 1993, and the model in Dean 1995). The authors observed that bilateral inactivation of cerebellar nuclei reduced eye speed, hence shifting the main sequence speed-amplitude relationship to lower

levels (see Figures 12, 13 in Robinson 1993 and Figure 4 in Dean 1995). We also know from Robinson 1980 that subjects with intact cerebellum restore their main sequence relationship, following a change in oculomotor dynamics (eye weakening). Nonetheless, we recognize that in Robinson's experiments it was not clear if the cerebellum influenced peak speed is because of updated motor plans in upstream regions such as superior colliculus or due to internal adaptive properties by means of STDP (although see Quessy et al., 2010 showing that the SC activity is not altered during saccade adaptation in the experiment's timescales).

To communicate our hypothesis on how the cerebellum influences peak speeds, in the manuscript, we added a figure showing the relationship between peak speed, duration and target amplitude. The simulations show that the heterogeneous plasticity version produces healthy saccades with preserved main sequence amplitude-speed relationship observed in monkeys (Fuchs 1967), while the other version produced relatively slower movements due to the inability to increase the peak speed with training. (the plot is added in reviewer #2 response #3 below).

Addressing the question of whether there is any behavioral evidence of an increase in peak speed during adaptation experiments:

Indeed, in classical saccade adaptation studies, there has been very little evidence showing that the adaptation is followed by an increase in peak speed, but recent behavioral experiments found evidence for this previously unexplored phenomenon. It was already reported in Chen-Harris et al., 2008 that the early and late components of the eye trajectory exhibit different patterns of change during adaptation. Specifically, Orozco et al., 2021 found that during the saccade adaptation tasks, not only is the endpoint error reduced across adaptation trials but also the acceleration period motor commands (that determine the peak speed) show trial-by-trial adaptation, independently from the decelerating period motor commands (influencing the endpoint errors). Such changes in acceleration period motor commands, can lead to trial by trial changes in peak speed.

In Orozco et al., 2021, the authors indicate that the neural basis of adaptation in acceleration period motor commands is still unknown, while the adaptation in deceleration period motor commands can be explained by the complex spike-driven/error-dependent plasticity in the PC

populations (Herzfeld et al., 2018). We noticed in PC recordings from (Soetedjo et al., 2008 and Herzfeld et al., 2018) that the PCs show characteristic pauses in complex spike activity throughout the movement, which can in principle result in higher levels of LTP proportional to movement duration. The CSpike pauses in Soetetdjo 2008 were very consistent across movements and occurred throughout the movement, although it was unknown what functional role they could serve; unlike the well-known function of error-dependent CSpikes occurring at the end of the movement (Herzfeld et al., 2018). As we show in our model, indeed the increased LTP can increase peak speed, while the LTD dominates to achieve reduced errors when the endpoint errors are high.

To address the reviewer's concern, we have added the following paragraph in the introduction as a rationale for increase in peak speed during saccade adaptation, Lines 101 – 112 in the tracked version:

"…. Studying if and how the cerebellar plasticity can jointly influence the peak speed and error is important, as recent behavioral experiments indicate that during saccade adaptation, the early and late components within a given movement are independently adjusted at different timescales (25,26). Such independent changes in early and late period motor commands can cause distinct changes in peak speed and visual error. However, the neural correlates of independent trial-by-trial adjustments of motor commands within a single movement are unknown. Notably, the activity in superior colliculus that is generally considered to convey a movement plan as input to the cerebellum (19), is shown to be unaltered across saccade adaptation trials (27). In principle, the cerebellum can leverage its adaptive filter property (28–31), where multi-timescale changes in synaptic weights are used to alter the cerebellar output based on measurements of movement quality, while its input pattern is unaltered."

<u>Regarding the question of why peak speed is increasing while the amplitude decreases:</u>

In the figures shown, the peak speed starts increasing slowly, only after the amplitude is reduced sufficiently closer to the target amplitude. i.e., in Figure 2f the peak speed starts to increase only after ~40 trials, where the amplitude is already reduced close to 10-degree. Notably, before the start of the increase in speed at ~40 trials, the movements were much slower

(<400deg/sec for 10-degree amplitude) than the typically high-speed movements observed in healthy conditions in monkeys (~500-550 deg/sec) (Fuchs 1967). Such healthy movements are restored only when we used heterogeneous plasticity rules (also see new Figure 3 in the updated manuscript). Our intention was also to show that only a cerebellar model with heterogenous PF-PC plasticity can account for increase in speed during training (when the acerebellar circuit saccade contribution is unchanged across trials e.g., from superior colliculus and brain stem burst). The error-dependent plasticity tends to correct the movement errors by producing a marked reduction in motor output, decreasing the peak eye speed to decrease the overshoot. Hence, the eye speeds will be relatively slower when using only the error-dependent plasticity, as the endpoint error information does not convey the quality of the movement in terms of peak speed. Once the errors are reduced, then the error-dependent plasticity is reduced allowing LTP to be dominant. LTP is proportional to movement duration as it persists throughout the movement duration while the CSpike firing does not increase, so that the slower the movements the more LTP is produced. This increases the burst PC activity and consequently the acceleration drive, resulting in higher peak speed. Note that such trial-by-trial changes in motor commands are controlled by the STDP updates of PF-PC synapses in the model, while the total input drive to the cerebellum through MF activation remains unchanged across trials. Such a separate treatment of STDP rules while keeping the MF consistent, shows that the cerebellum is not completely dependent on the motor plans communicated by upstream regions for velocity control (more discussion about this in the Reviewer question #2 below).

In summary, it is known that the cerebellum is necessary for maintaining the normal relationship between amplitude and peak speed (Robinson 1993, Robinson & Opstal 1980). While behavioral evidence recently discovered acceleration period motor drive increases during adaptation tasks (Orozco 2021). In this context, we intended to find how the cerebellum accounts for increase in peak speed (with MF firing pattern remaining constant across trials) and studied the STDP mechanisms that can potentially explain this effect. We showed that only dual STDP model is capable of increasing the peak speed during training.

We added the following paragraph in the discussion section (Lines 492-507) to highlight this:

"In (25,26) the authors showed that during saccade adaptation to abrupt target shifting, the new eye trajectories exhibited different patterns of change in early and late movement periods (corresponding to the acceleration and deceleration phase, respectively). If different timescale processes were used to adjust the motor commands for acceleration and deceleration periods within a movement, then one might expect dissociation between changes in peak speed and end displacement (and hence end movement error). Recently in (25), the authors showed that the motor commands in the acceleration period change relatively slowly compared to those in the deceleration period across saccade adaptation trials. A slower change in the accelerating motor command can produce slower increase in peak speed, while a faster change in decelerating command can ensure that the saccade movement errors are quickly minimized. Our model predicts that the changes in peak speed can be mediated by error-independent LTP, which produces synaptic changes at a slower time-scale compared to error-dependent LTD. Open questions for network modeling are now whether and how different plasticity mechanisms within the cerebellar circuit could explain the systematic decay of adapted motor commands during pauses/breaks between different blocks in the experiment and error clamp conditions as observed in (25)."

(c) The authors describe that these velocity increases demonstrated that the movement is 'optimized' or 'improved', but they do not explain what the cost function or metric of improvement is. Overall, it would have been helpful to initially lay out the expected model behavior regarding error and peak velocity and the underlying rationale/evidence.

We used the term 'optimized' or 'improved' with respect to the ecological objectives of saccades, i.e., to reach the target as fast and as accurately (with little error) as possible. This can be looked at from the perspective that an organism will gain an advantage if it manages to quickly locate, process, and exploit useful information through rapid sensing and fine motor control. Regarding the cost function, several variants can be used to describe the metric of improvement during saccades. For example, initially, the model in Harris & Wolpert 1998 assumed that saccades minimized the endpoint variance, while there were constraints on motor commands due to signal-dependent noise. But in later studies, (Chen and Shadmehr 2008), the authors used cost function that involved continuous kinematic trajectory and motor commands, while explicitly penalizing higher movement duration. Although seemingly different, the above

mentioned cost functions ultimately describe a common goal i.e., to move as fast as possible, limiting the accumulation of movement errors that might occur due to speed-accuracy tradeoff. In Saeb et al., 2011, the authors use a cost function that consists of a kinematic trajectory error term, and a penalty on high motor/muscle activation in the context of head-free gaze shifts. Even this cost function produces optimal saccades under head-free condition, but the cost on error trajectory is biologically infeasible due to sensory suppression during saccades. As elegantly pointed out in (Harris 1998), it is important that the cost functions use terms that corresponds to whether the underlying neural circuit is privy to the particular information that the cost is based upon or not. In Harris 1998, the author states that the movement time might not be explicitly available to the cerebellum raising a puzzle on how the cerebellum can optimize the movement duration. Hence in our previous cerebellum-inspired robotics work (Kalidindi 2020), we introduced a new cost function that is similar to that of Chen 2008, but also more realistic as it works with the information that the cerebellum has access to (namely endpoint errors and cumulative Purkinje cell activity). We used a gradient descent method, and concluded that some kind of error-independent plasticity mechanisms should exist to account for the improvement in movement speed, as a detailed temporal error trajectory was unavailable. In summary, multiple cost functions can reproduce the optimality observed in saccades.

Overall, one of the main results of the cost function approaches, irrespective of the exact type of formula, has been to show that the saccades maintain a balance between speed and accuracy. So, we considered that the faster the movements can be made without incurring errors, the more optimal they are and hence used the terms “optimized” and “improved”. Indeed, in figure 2f we show how once the amplitude becomes constant at around 10 degrees, the eye speed continuously increases (after ~40 trials).

We did not use any explicit cost function to tune the cerebellar synapses in the submitted manuscript. The focus here, was to understand how, in a realistic bottom-up neural circuit, optimal saccades emerge (with the experimentally observed speed-amplitude relationship) from the internal network mechanisms. Eventually, this sort of “intrinsic” optimization must lead to some ecological advantage (faster and accurate), without formulating the optimal control problem with explicit cost functions.

We agree that using the term “optimization” without mentioning cost functions can be ambiguous. But providing a detailed description of what we mean by optimality is increasing the size the introduction, without contributing much to the increase in clarity to the results section. To avoid any confusion about the cost functions to the reader, we removed any references to optimality/improved from the introduction. Instead, we used specific terms such as increase in peak speed, and decrease in visual error. However, we emphasized the different considerations of optimality in the discussion section.

In the discussion we dedicated a paragraph to describe different cost functions that can emulate biological saccade characteristics, and how our model can control the quality of saccades using the bottom-up STDP principles (Lines 534-565). We further show that the heterogenous STDP rules explain the production of saccades that are closer to main sequence peak-speed vs amplitude relationship (please refer to our reply to reviewer #2 question #3).

R2 – Q2. The study suggests that the cerebellum plays a role in planning and dictating saccade peak velocity. This is not in contrast to current evidence suggesting that the cerebellum implements a plan determined by the superior colliculus. It would be helpful for the authors to clarify and expand upon the rationale for cerebellar control of saccade peak velocity.

Thank you for raising this important question. Indeed, we do not disagree with the view that the cerebellum can implement a movement plan determined by the superior colliculus, as there is evidence that a part of the incoming MF activity correlates with SC burst patterns (MF afferents arising from NRTP, Ohtsuka and Noda 1992 and van Kan et al., 1993) and that the SC burst patterns correlate with eye velocity within a trial (vanOpstal 2011). However, given that the PF-PC synapses undergo trial-by-trial adjustments, it is possible that even with the same SC-drive, the PC output can be modulated owing to the adaptive filter property of the circuit (Dean, Porrill and Jorntell 2010). Thus, a change in movement can be due either to trial-by-trial changes in the SC drive to the cerebellum or to changes in cerebellar output (with unchanged SC drive). In earlier studies, researchers did not find evidence for trial-by-trial changes in locus of activity in the SC with changes in saccade displacement during target jump tasks, indicating that the adaptation occurs downstream of SC circuit (Quessy JNeuro 2010, Frens & Opstal 1997,

although see Takeichi, Fuchs 2007). Important to our current article, in a recent study (Orozco 2021), the authors show that during saccade adaptation, early and late period motor commands during a movement undergo independent changes in a trial-by-trial manner. Specifically, the late period motor commands are updated rapidly within a few trials, while the early period motor commands are updated in a relatively slower time scale. The changes in early period motor commands will lead to trial-by-trial change in peak speed, while the late period motor commands determine the movement error.

It is currently unknown experimentally if the locus of SC activity changes in a trial-by-trial manner to represent the changes in peak speed (or early period motor commands) across trials.

Given that (1). The cerebellum is important for maintaining high peak speed in inactivation experiments (Robinson 1993), (2). The cerebellum is supposed to operate as an adaptive filter (with multi-timescale plasticity mechanisms) allowing updating its output patterns at heterogenous time scales for any fixed input pattern, and (3) there is no evidence that trial-by-trial changes in SC activity correlate with trial-by-trial changes in eye speed (Quessy et al. 2010) we sought to answer if the cerebellum can increase the peak speed through its STDP mechanisms, while the target-related input drive to the cerebellum and the rest of the control circuit remains constant across trials.

Overall, our objective is not to propose that the cerebellum always performs velocity control independent of the superior colliculus drive. Indeed, we show that modifying the input drive to the cerebellum (by MF modulation) influences the PC activity and movement speed (in Figure 3 of the old draft, and Figure 4 of the revised draft). Our emphasis was to shed light on STDP mechanisms that enable peak velocity control without necessarily requiring a variable input drive from the superior colliculus.

To make this point more explicit, we added a paragraph at the beginning of the results section in the updated manuscript (Lines 187-193):

"There are two main ways by which the given saccade controller can modulate the saccades. First, STDP mechanisms influence the strength of PF-PC synapses such that the PC population activity is modified. In this case the total input MF drive to the cerebellum from the

superior colliculus can remain unchanged across trials. Second, the modulation of neural activity occurs upstream of the cerebellum (e.g., in the superior colliculus), and influences the cerebellum to produce a different PC output. Here, we consider the former process focusing on the effect of STDP on end errors and peak speed."

R2 – Q3. A suggestion to help clarify hypotheses and validate the model would be to demonstrate that the model can simulate the main sequence. If this has been shown in an earlier paper, then a statement as such would suffice.

We thank the reviewer for this suggestion. In the updated manuscript, we have added the plot (attached below) depicting the relationship between amplitude and peak speed emerging from the simulations. The figure shows that the simulations with dual plasticity terms reproduce the levels of peak speed observed in monkeys at different amplitudes (while circuit properties including the input from the superior colliculus, MF activation, and brain stem bursts, remain unchanged). As a reference to monkey experiments, we are including the snapshot of monkey kinematic characterization from Fuchs 1967 here (Figure 5 from Fuchs 1967).

Note that the training has been carried out by presenting the targets randomly in each trial. We also added a figure depicting the learning curves (Supplementary Figure 2) where the initial saccades to the mentioned targets incur endpoint errors. We can see that the only-LTD movements cannot increase the eye speed, due to the inability to potentiate the PF-PC synapses and produce a higher PC activation at a given input activation level.

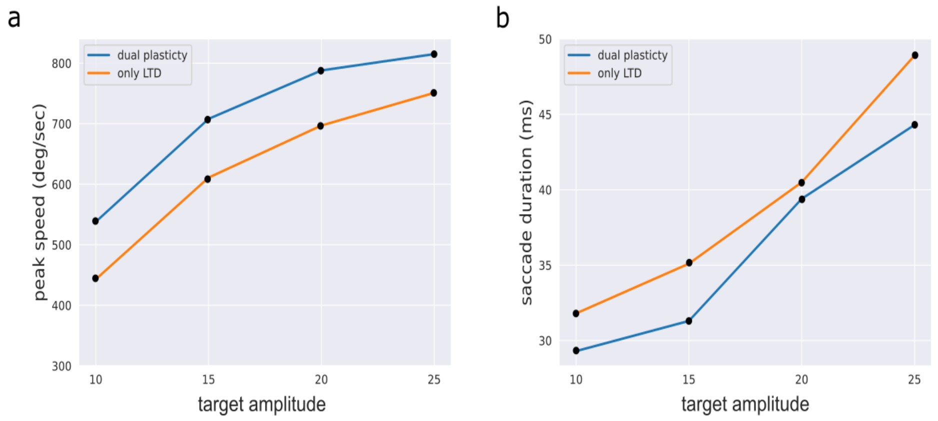

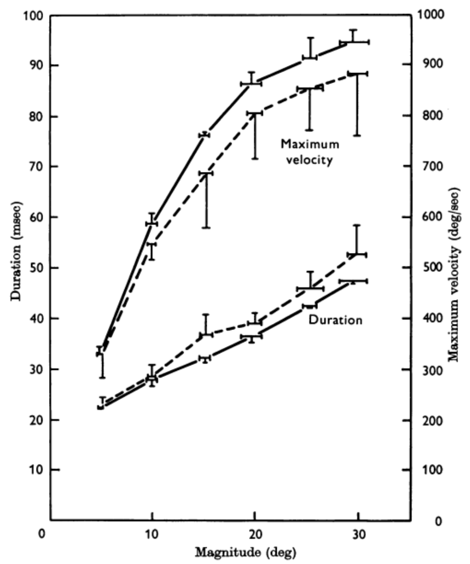

Fig. 5. Duration and maximum velocity of horizontal saccades against magnitude. Each point is the mean of at least twenty-four observations, eight from each monkey; the bars represent the standard deviations from the mean. —, Nasal; - - -, temporal.

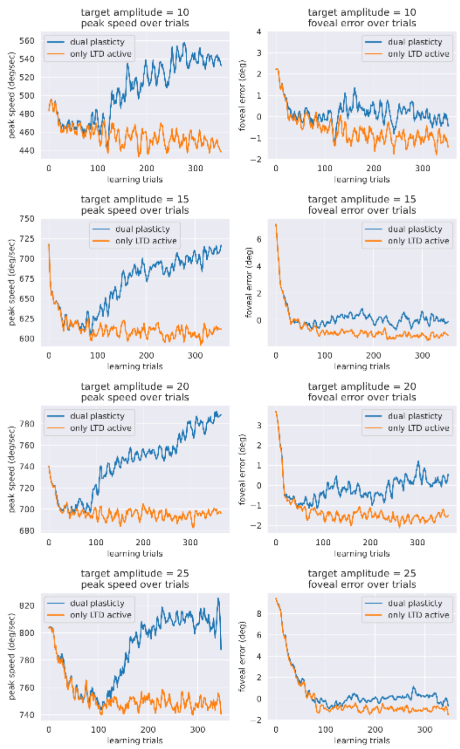

Figure from Fuchs 1967 (Monkey saccades)

R2 – Q4. A lesser point is the use of the terms 'vigor' and 'accuracy'.

a. Vigor is usually described as peak velocity normalized to movement amplitude. This allows

one to distinguish between a change in peak velocity due to reward from the well-established change in peak velocity due to movement amplitude. The authors use the terms interchangeably here, but I recommend using only peak velocity unless they are normalizing to the movement amplitude.

b. Accuracy is often used to describe the variability of movement endpoints over many trials and is thought of in the context of speed-accuracy trade-offs in movement. I understand that the single-trial overshoot described in this paper is also a reflection of accuracy, but it is more akin to the error observed in a movement adaptation study than in a speed-accuracy study. Thus, to improve the clarity, it would be helpful to explain that error, overshoot, and accuracy are all the same here and to define what is meant by them.

a. We agree with the reviewer that the terms 'vigor' might be confusing given their use in specific task protocols that include the study of reward-modulated peak speed. Hence, in the updated manuscript we replaced this term with peak speed.
b. Thank you for pointing out this confusion. We replaced the term accuracy with end error where necessary in the manuscript, and provided definitions for terms such as overshoot/undershoot. Furthermore, we thoroughly checked for potentially ambiguous terms in the manuscript and either replaced them with precise descriptions and/or we provided definition for each such term.

Reviewer #3:

Fruzzetti et al. propose a biologically realistic model of the cerebellar computational dynamics. Importantly, the manuscript demonstrates the potential mechanisms by which the cerebellum can leverage heterogeneous spike-timing-dependent plasticity (STDPs) to optimize oculomotor adaptation.

The authors have done a great job setting up the research question and the motivations for developing the proposed model. I also especially find the introduction and discussion to be interesting and informative. The primary questions I have regarding the manuscript

concern the modeling choices adopted and the statistical analyses performed. Below, I have included specific concerns that I hope the authors could help address.

Thank you very much for the positive evaluation of our work and for the helpful suggestions. The reviewer's comments have been very helpful in improving the manuscript, and below we addressed the specific concerns from the reviewer.

R3 - Q1. I believe the proposed modelling schemes and the learning rules are intriguing and innovative. However, it would be helpful for the authors to consider adding citations and more justifications for specific modelling/parameter choices implemented in the proposed model. For example, the authors chose to use the voltage of the deep cerebellar nuclei (rather than the average firing rates) to determine the cerebellar contribution (yc). Could you please provide additional information regarding this modeling choice? Additionally, could the authors discuss how this affects the overall observed model dynamics? Additionally, how would the model dynamics and conceptual interpretation of the observed dynamics change if the average firing rates were used instead?

We thank the reviewer for this suggestion.

Our goal was to identify the contribution of the PC populations in encoding and controlling saccades, in isolation from other areas such as the superior colliculus, brainstem, and even the deep cerebellar nuclei. This is the main reason we also simulated a rate-based brainstem circuit and not a spike-based one, and avoided a spike-realistic model of the superior colliculus. If we were to use firing rates of DCN instead of voltages, we would have needed to add another filter to convert the DCN-spikes into average firing rates to communicate with the brainstem. This essentially will likely lead to the same results after tuning the spike-to-rate conversion step. Notably, the model can replicate both PC activity and the saccade kinematics even by eliminating the DCNs and generating the brainstem drive by integrating the PC subpopulation activity at a dummy summation node. To clarify this, we have added additional rationale in the methods. Moreover, we wanted to understand if we could emulate the observed characteristics of the saccades with as simple DCNs as possible, that simply relay the PC information to the

brainstem circuit. We clarified the idea behind simplifying the DCN circuit in the updated methods in Lines 754-764 (in the tracked version) as follows:

"In the model, PCs inhibit DCN, which are the output of the cerebellum. In our work cerebellar contribution ("yc" in equation 3.0) is determined by the voltage of DCN. We preferred to use the voltage of the DCN instead of computing DCN average firing rates. This is useful for two reasons. First, we can focus on whether the PC output is sufficient to modulate eye speed, and end displacement without adding complex spiking properties in the DCN circuit. Second, by avoiding spike-to-rate transformation at the DCN level, we increase stability of the simulations and reduce the effort for hyper-parameter tuning. The output is derived by subtracting the DCN basal voltage, (obtained by running the cerebellar simulation, without inputs, for 500 ms) to the current DCN voltage. We do not expect the results to change significantly even if we add spiking property to the DCN's provided the DCN's transmit the PC activity reliably to the brain stem burst neurons in terms of average firing rate. …"

Details of parameter selection is presented in reviewer #3 response 4.

R3 - Q2. Regarding the plasticity rule, are there competing theories for what the plasticity rule should be for a cerebellar system? It will be helpful for readers to understand the limitations of the model dynamics if the relevant context of other possible plasticity rules is provided.

Indeed, there are other potential plasticity rules, that can perform the similar function described in our simulations. In our simulations, we have used LTP and LTD at the level of PF-PC excitatory synapses. Additionally, there is also an indirect pathway through inhibitory molecular interneurons (MLIs) with reciprocal plasticities compared to the direct PF-PC pathway (Michiel et al., 2015). The redundancy of plasticity mechanisms in the cerebellar cortex is crucial especially in case of lesions, when one mechanism can compensate for the lack of the other; here, we wanted to investigate physiological conditions and the PF-PC plasticity proved sufficient to reproduce experimental observations. On the other hand, an important gap in knowledge arises from the fact that the DCN circuitry in the cerebellar system also features multiple plasticity processes at MF-DCN and PC-DCN connections, occurring at slower time scales. In this study, we have simplified the DCN circuitry to transmit the inhibitory PC

population activity to the brain stem burst generator. However, given the complexity of the DCN circuitry, it can potentially show a more dominant role than what is considered in the current work. We know that motor memories although rapidly formed at the level of PF-PC synapses, eventually transfer to the nucleus site (example VOR/OKR inactivation experiments, (Monaco et al., 2014; D'Angelo et al., 2018).

Another potential factor underlying the increase in peak eye speed could reside in changes happening upstream of the cerebellum e.g., in the superior colliculus. There is still not experimental evidence that examines if the SC activation changes across trials correlate with the changes in peak speed across saccade adaptation trials (Orozco 2021). On the contrary it was shown that the locus of SC activity does not change with saccade adaptation (Quessy et al., 2010).

We added a discussion about the alternative PF-MLI-PC plasticity in Lines 486-491, as follows.

"In this work, we explored the function of two among multiple plasticity mechanisms in the cerebellar microcircuit (53,54). Even the PFs project onto the PCs through two different pathways, direct excitatory PF-PC connections and di-synaptic inhibitory PF-MLI-PC connections (55,56). In the presented simulations, we considered the PF-MLI-PC synapses to be fixed and simulated the effect of STDP in direct PF-PC connections only. We expect that similar effects can be reproduced by means of PF-MLI-PC plasticity."

Furthermore, we added the discussion about the potentially complex role of DCN connections, plasticities in the new limitations paragraph of the discussion (Lines 600-627), as follows.

"The limitation of the current model is the simplicity of different brain regions, in spite of a detailed biologically inspired construction of the cerebellar network. The model focused on how the PC population activity can be adaptively modified to influence peak speed, and end visual error while the target-related input drive to the cerebellum and the rest of the controller remains unaltered across trials. But the saccade control is composed of different regions, and especially Superior colliculus (SC) activity is implicated in determining a motor plan, that the downstream cerebellum and brainstem burst neurons can implement (73,74). In principle, the SC activity can be altered to change the input drive (MF activity) to the cerebellum and the brain

stem burst neurons, which can subsequently lead to change in peak eye speed. Notably, our modeling results do not negate the possibility that the SC influences peak eye speed by modulating its input MF drive to the cerebellum. Indeed, in Fig 4, we show that modulating the input MF drive to the cerebellum results in different peak eye speeds. However, the set of presented results are better viewed as evidence that the cerebellar STDP mechanisms can exert peak speed modulation, even if the SC/target-related input drive remains constant (see (27)). Furthermore, our future work will focus on implementing a more realistic DCN circuit. Each DCN is a hub of diverse incoming projections from MFs, PCs, and also a recurrent nucleo-olivary inhibition loop (75). Biological DCNs have additional connections from MFs and the nucleo-olivary loop that are not included here, potentially limiting the predictive capabilities of our model. Functionally, the DCNs can display unique spiking characteristics such as rebound spikes during saccades (22), that can help them sustain motor output while the inputs are extinguished. The DCN-IO circuit is important to generate internal spatio-temporal dynamics coordinating large sets of PCs (46) and to regulate plasticity in the circuit by adding slower time constants to the learning process (40,53,76). These aspects should become critical in complex multifactorial behaviors evolving over multiple spatial scales but, since this was not the case here, the absence of a more precise representation of the IO-DCN loop was unlikely to cause relevant drawbacks. Overall, it remains to be seen how the cerebellum model presented in this work shares computations with other regions, by integrating realistic circuit models of SC, DCN, and brainstem circuits."

The alternative SC mechanism underlying eye speed changes are also included in the paragraph on limitations mentioned above.

R3 - Q3. Information regarding statistical analyses appears to be missing. What kinds of statistical tests were performed to evaluate the model dynamics (e.g., the behavioral and neural effect of cerebellar learning by the PF-PC plasticity mechanisms) and what were the results? Such information is important for readers to understand different aspects of the models and their emergent behaviors. For example, it is unclear if the cerebellar spiking neural network learning (as induced by the dual plasticity) led to a significant improvement in accuracy and vigor across multiple movements over time.

We thank the reviewer for suggesting this important detail. We updated the manuscript with statistical descriptions where necessary. In Lines 353-366 we added:

"To compare the effect of input level on the maximum population activity and maximum speed, two separate one-way ANOVA analysis were performed. Each input level had 10 separate saccades towards the 10-degree target (while the plasticity was inactivated to have identical conditions of movement). The one-way ANOVA revealed that there is a significant statistical difference in the maximum PC population activity across input levels ($F(3, 36) = 103.15$, $p < 0.001$), and similarly a significant statistical difference in the peak speed across input levels ($F(3, 36) = 133.26$, $p < 0.001$). Post hoc comparisons were used to contrast the maximum of the population activities and the maximum speed obtained with the different input levels. For population activity, significant differences were found between each pair of input levels ($p<0.001$ after Bonferroni correction) except between input level = 1.0 and input level = 0.83 ($p=0.18$ after Bonferroni correction). For the maximum speed, significant differences were found between each pair of input levels ($p<0.01$ between input-level = 1.0 and input-level = 0.83, and $p <0.001$ for remaining pairs after Bonferroni correction)."

In Lines 390-392, and Lines 393-395 we added:

"($p <0.001$ with t-test, considering the maximum burst activity in 10 trials each before and after training; normality was verified for both groups of the data using kstest)."

In Lines 403-410, we added:

"Switching off LTD caused opposite effects on the activity of burst and pause PC subpopulations, compared to those observed when LTP was switched-off. The activity of burst PCs increased above the baseline (Fig 5e) ($p <0.001$ with t-test, considering the max activity in 10 trials each before and after training; normality was verified for both groups of the data using kstest). On the other hand, the activity of pause PCs decreased towards the baseline (Fig 5f) ($p <0.001$ with t-test, considering the minimum activity in 10 trials each before and after training; normality was verified for both groups of the data using kstest)."

In Lines 424-429 we rewrote the description:

"As expected, the burst PC subpopulation firing rate remained constant (Fig 6a) without statistical significance (p = 0.16 with t-test, considering the maximum activity in 10 trials each before and after training; normality was verified for both groups of the data using kstest). Pause PC subpopulation firing rate showed statistically significant modulation during the training (Fig 6b) (p <0.001 with t-test, considering the minimum activity in 10 trials each before and after training; normality was verified for both groups of the data using kstest)."

In Lines 435-441, we rewrote the description as:

"As expected, when the dual-plasticity is switched-off at the PC pause subpopulations the population firing rate remained constant (Fig 6f) (p = 0.23 with t-test, considering the minimum activity in 10 trials each before and after training; normality was verified for both groups of the data using kstest). However, the burst PC subpopulation firing rate decreased during training (Fig 6e) (p <0.01 with t-test, considering the max. activity in 10 trials each before and after training; normality was verified for both groups of the data using kstest)."

R3 - Q4. The authors discussed tuning the learning rate parameters of LTP and LTD as an essential step toward generating optimal behavior, especially in the context of both burst and pause subpopulations. However, it is unclear how such tuning was performed in the present manuscript. Please consider including additional information regarding the different steps taken to ensure optimal tuning. Additionally, could the authors discuss the mechanisms by which this tuning process is achieved in nature?

LTP and LTD parameters were set based on the values in previous studies where the same cerebellar model was challenged in simulating different tasks, while fine tuning was carried out using gross exploration and binary search.

We updated the methods section with further details of parameter tuning. We added a detailed description in the following paragraph in Lines 895-927:

"We found a range of $(\alpha_{PC_b}, \beta_{PC_b}, \alpha_{PC_p}, \beta_{PC_p})$ *parameters that led to increase in peak speed and decrease in foveal error through a guided binary search. In the first step, we set only two*

parameters values, LTP and LTD (or α_{PC} and β_{PC}), commonly across burst and pause PC subpopulations. These parameters were selected from multiple solutions of LTP and LTD values found in (82) on different sensorimotor tasks. Note that these initial parameters were not fine-tuned for the saccade task, but were well tested in other sensorimotor tasks (82). Notably, even without fine tuning, a large range of parameter values were observed to modulate atleast the foveal error. Subsequently, for finding the optimal parameter tuning that influence both movement speed and error, we performed a search in the vicinity of the initial parameters (testing the parameter changes in the range of [10^{-9}-10^{-4}] for α_{PC_b} and α_{PC_p}, and [10^{-9}-10^{-4}] for $\beta_{PC_b}, \beta_{PC_p}$). Note that while changing LTP parameter, the LTD parameter was held constant, and vice-versa so that a set of parameters that can influence error and speed was found. We followed two simple steps to evaluate the quality of parameter selection. First, we visually verified if the selected parameters produced information flow from MF input activation to the PC output. i.e., we verified that the norm of PC population activity was different from the baseline and the SSpikes persisted at least until as long as the MF activity persisted. Second, we evaluated the parameters by running the saccades to 10-degree target for multiple trials, and considered the parameters as a good set if the foveal error after convergence of training was ~0.5 degree, and if the peak speed was higher at the end of 150-200 trials was higher than the first 10 trials. We manually selected a subset among the parameters found by binary search and used them for simulations (Supplementary Table 2). In Fig 1b, we visualized the distinct effect of the learning rule with exemplary learning rate parameters on the activity of burst and pause PC subpopulations. The change in PF-PC weights ($\Delta W_{PF_i\,PC_j}$) of a given PC subpopulation depended on the ratio between the two learning rates (α_{PC_j}, β_{PC_j}) and on the foveal error occurring after each saccade.

As a result of the tuned learning rates in the simulations, the sign of $\Delta W_{PF_i\,PC_j}(t)$ for PC pause and PC burst were concordant if the error was equal to 0.0 or higher than 1.0. Conversely, the weight changes, $\Delta W_{PF_i\,PC_j}(t)$, in pause and burst subpopulations were discordant if the error was around 0.5 deg. In the discordant zone PF-PC connections of pause subpopulations decreased their excitatory weights due to a dominant LTD process (increasing the amount of drop in firing rate below baseline), while PF-PC connections of the burst subpopulations

increased their excitatory weights due to a dominant LTP process (increasing their firing rate above baseline).”

Additionally, by following your suggestion, we have added a paragraph in the discussion section with a brief description of the biological mapping of learning rate parameters as follows (Lines 928-936):

“The mechanistic description of long-term plasticity changes that we have used is relatively simple and as previously used in several protocols where the cerebellum is crucially involved in generating motor adaptation (41,42,66,82,83). However, it is difficult to map the learning rate parameters to specific biological components. For instance, in glutamatergic synapses, LTP and LTD are produced with a change (increase and decrease, respectively) in the number of AMPA receptors present in the post-synaptic density (84). These changes are regulated by complex intracellular chain reactions involving multiple proteins. Each step of these processes has different timings and dynamics which are, in the end, responsible of the precise and differential time course of LTP and LTD.”

R3 - Q R3 - Q 5. What are the technical limitations of the proposed model that the readers should be aware of/take into account as they interpret the observed results?

The main limitation of the proposed model is the simplicity of different brain regions, in spite of a detailed biologically inspired construction of the cerebellar network. Our modeling was focused on explaining how the PC population activity can be adaptively modified to influence peak speed and movement error, while the target-related input drive to the cerebellum and the rest of the controller remain constant. In the real case, the saccade control system is composed by different regions. In particular, activity in the superior colliculus is correlated with the temporal pattern of eye speed (Opstal 2011), and a spiking neural network model of SC could indeed emulate saccades without simulating the cerebellar circuit (Kasap et al., 2017). In principle, the SC activity can be adjusted across trials to change the peak speed, by changing the input drive both to the cerebellum (through NRTP-MF afferents) and the brainstem burst neurons. Recordings from SC suggest in fact that the locus of activity in the SC remains unaltered during saccade adaptation (Quessy et al., 2010). Our modeling results provide evidence that the

cerebellar STDP machinery can exert control on peak eye speed, even if the SC/target-related input drive remains constant. Another limitation is that the DCN itself is a hub of different projections coming from MFs, PCs, and recurrent nucleo-olivary inhibitory loops. The DCNs are hypothesized to display unique spiking characteristics during saccades (Gad 2010), that help them sustain motor output during receding inputs. Overall, it remains to be seen how our cerebellar model shares computations with other regions when realistic circuit models of SC, DCN, and brainstem circuits are integrated.

We added another paragraph in the discussion (Lines 600-627) to highlight the limitations, as already added to reviewer #3 response #2:

References:

1. Fuchs, A. F. (1967). Saccadic and smooth pursuit eye movements in the monkey. *The Journal of Physiology*, *191*(3), 609.
2. Optican, L. M., & Robinson, D. A. (1980). Cerebellar-dependent adaptive control of primate saccadic system. *Journal of neurophysiology*, *44*(6), 1058-1076.
3. Robinson, F. R., Straube, A., & Fuchs, A. F. (1993). Role of the caudal fastigial nucleus in saccade generation. II. Effects of muscimol inactivation. *Journal of neurophysiology*, *70*(5), 1741-1758.
4. Harris, C. M., & Wolpert, D. M. (1998). Signal-dependent noise determines motor planning. *Nature*, *394*(6695), 780-784.
5. Harris, C. M. (1998). On the optimal control of behaviour: a stochastic perspective. *Journal of neuroscience methods*, *83*(1), 73-88.
6. Takeichi, N., Kaneko, C. R., & Fuchs, A. F. (2007). Activity changes in monkey superior colliculus during saccade adaptation. *Journal of Neurophysiology*, *97*(6), 4096-4107.
7. Chen-Harris, H., Joiner, W. M., Ethier, V., Zee, D. S., & Shadmehr, R. (2008). Adaptive control of saccades via internal feedback. *Journal of Neuroscience*, *28*(11), 2804-2813.
8. Quessy, S., Quinet, J., & Freedman, E. G. (2010). The locus of motor activity in the superior colliculus of the rhesus monkey is unaltered during saccadic adaptation. *Journal of Neuroscience*, *30*(42), 14235-14244.
9. Gad, Y. P., & Anastasio, T. J. (2010). Simulating the shaping of the fastigial deep nuclear saccade command by cerebellar Purkinje cells. *Neural networks*, *23*(7), 789-804.
10. Saeb, S., Weber, C., & Triesch, J. (2011). Learning the optimal control of coordinated eye and head movements. *PLoS computational biology*, *7*(11), e1002253.
11. Goossens, H. H. L. M., & van Opstal, A. J. (2012). Optimal control of saccades by spatial-temporal activity patterns in the monkey superior colliculus. *PLoS Computational Biology*, *8*(5), e1002508.
12. Kasap, B., & van Opstal, A. J. (2017). A spiking neural network model of the midbrain superior colliculus that generates saccadic motor commands. *Biological Cybernetics*, *111*(3), 249-268.
13. Kalidindi, H. T., Vannucci, L., Laschi, C., & Falotico, E. (2020). Cerebellar adaptive mechanisms explain the optimal control of saccadic eye movements. *Bioinspiration & Biomimetics*, *16*(1), 016004.

14. Orozco, S. P., Albert, S. T., & Shadmehr, R. (2021). Adaptive control of movement deceleration during saccades. *PLoS computational biology*, *17*(7), e1009176.

15. D'ANGELO, E. G. I. D. I. O. (2011). Neural circuits of the cerebellum: hypothesis for function. *Journal of integrative neuroscience*, *10*(03), 317-352.

16. Monaco, J., Casellato, C., Koch, G., & D'Angelo, E. (2014). Cerebellar theta burst stimulation dissociates memory components in eyeblink classical conditioning. *European Journal of Neuroscience*, *40*(9), 3363-3370.

17. Antonietti, A., Monaco, J., D'Angelo, E., Pedrocchi, A., & Casellato, C. (2018). Dynamic redistribution of plasticity in a cerebellar spiking neural network reproducing an associative learning task perturbed by TMS. *International journal of neural systems*, *28*(09), 1850020.

18. D’Angelo, Egidio, Lisa Mapelli, Claudia Casellato, Jesus A. Garrido, Niceto Luque, Jessica Monaco, Francesca Prestori, Alessandra Pedrocchi, and Eduardo Ros. "Distributed circuit plasticity: new clues for the cerebellar mechanisms of learning." *The Cerebellum* 15, no. 2 (2016): 139-151.

19. Smith, M. A., Ghazizadeh, A., & Shadmehr, R. (2006). Interacting adaptive processes with different timescales underlie short-term motor learning. *PLoS biology*, *4*(6), e179.

20. Michiel, M., Henk-Jan Boele, Jochen K. Spanke, Jan-Willem Potters, Katja Kornysheva, Peer Wulff, Anna CHG IJpelaar, Sebastiaan KE Koekkoek, and Chris I. De Zeeuw. "Evolving models of pavlovian conditioning: cerebellar cortical dynamics in awake behaving mice." *Cell reports* 13, no. 9 (2015): 1977-1988.
